# Approaches to the Structure-Based Design of Antivirulence Drugs: Therapeutics for the Post-Antibiotic Era

**DOI:** 10.3390/molecules24030378

**Published:** 2019-01-22

**Authors:** Nolan Neville, Zongchao Jia

**Affiliations:** Department of Biomedical and Molecular Sciences, Queen’s University, Kingston, ON K7L 3N6, Canada; n.neville@queensu.ca

**Keywords:** structure-based drug design, crystallography, NMR, modelling, docking, antibiotics, antivirulence drug

## Abstract

The alarming rise of multidrug-resistant bacterial strains, coupled with decades of stagnation in the field of antibiotic development, necessitates exploration of new therapeutic approaches to treat bacterial infections. Targeting bacterial virulence is an attractive alternative to traditional antibiotics in that this approach disarms pathogens that cause human diseases, without placing immediate selective pressure on the target bacterium or harming commensal species. The growing number of validated virulence protein targets for which structural information has been obtained, along with advances in computational power and screening algorithms, make the rational design of antivirulence drugs a promising avenue to explore. Here, we review the principles of structure-based drug design and the exciting opportunities this technique presents for antivirulence drug discovery.

## 1. Introduction

The use of chemotherapeutic agents to treat bacterial infections began with the sulphonamides and penicillin in the 1930s, marking one of the most significant medical breakthroughs of the twentieth century. The so-called golden era of antibacterial drug design followed suit, with the development of almost all of the antibacterial drugs in use today occurring between the 1940s and 1970s [1,2]. The widespread therapeutic use of these drugs, or agents derived from them, has contributed dramatically to global public health and an increase in life expectancy [3].

Unfortunately, widespread resistance to the drugs developed in the twentieth century has been emerging at an alarming rate. This problem is compounded by the failure to discover and develop new classes of replacement drugs. Antibiotic development has stagnated to such a degree that only eight of the 51 drug candidates currently in the clinical pipeline differ substantially from existing classes of antibiotics [4]. Indeed, antibiotic resistance now undermines the ability to treat many bacterial infections [5,6,7], and signals the dawn of a post-antibiotic era [8]. Clearly, the status quo of antibiotic development is not sustainable. 

Nearly all of the golden-era antibiotics were discovered via empirical whole-cell screening programs, which identify inhibitors that prevent bacterial growth—and thus target essential cellular processes such as cell wall synthesis (i.e., β-lactams) or DNA replication (i.e., fluoroquinolones). As such, traditional antibiotics impose enormous pressure for selecting resistant variants in a population, which often occurs within a few years of clinical use [9]. Additionally, antibiotics that target central growth pathways—which are common among pathogenic and non-pathogenic species alike—result in off-target perturbation of the microbiome that can open up new niches for colonization by resistant pathogens [10]. 

**Figure 1:**
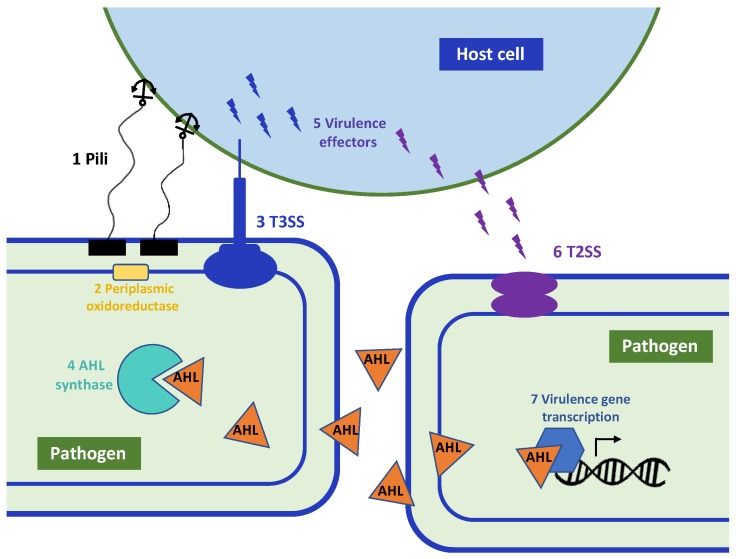
Overview of bacterial virulence pathways that have been targeted for inhibition via SBDD. Pathogen adhesion to the host cell; 1. Inhibitors of pili biogenesis and assembly (e.g., chaperone/usher pathway). Folding and disulfide bond formation in secreted virulence factors; 2. Inhibitors of periplasmic thiol oxidoreductase (e.g., DsbA). Virulence effector injection into the host cell; 3. Inhibitors of the type 3 secretion system (T3SS). Acyl homoserine lactone (AHL) quorum sensing signal generation; 4. Inhibition of AHL synthase (e.g., LasI). Effector protein toxicity; 5. Toxin neutralization to prevent host cell damage. Toxin protein secretion into the extracellular milieu; 6. Inhibitors of the type 2 secretion system (T2SS) to block toxin translocation across the outer membrane. Transcriptional activation of virulence genes in response to stress or quorum signals; 7. Inhibitors of AHL binding to cognate transcription regulators (e.g., LasR).

Antivirulence drugs have gained considerable research interest in the past decade as an alternative approach to traditional antibiotics [11]. Virulence—which is synonymous with pathogenicity—is classically defined as the ability of an organism ′to enter into, replicate within, and persist at host sites that are inaccessible to commensal species′ [12]. Antivirulence drugs disrupt the process of infection, but in contrast to antibiotics, they do not directly affect bacterial growth or viability. The earliest examples of the antivirulence approach include inactivation of bacterial toxins, such as tetanus and diphtheria [13,14]. More recently, a myriad of other virulence factors have been suggested as promising antivirulence targets (as reviewed by [15,16,17,18]). Some of the most attractive antivirulence strategies include inhibition of quorum sensing and virulence regulation [19], virulence factor secretion and function [20], and bacterial adhesion and colonization [21] (Figure 1). This ′disarm—don′t kill′ approach avoids some of the selective pressure imposed by antibiotics, potentially providing an evolution-resistant antimicrobial [22]. Additionally, since antivirulence drugs are specific to pathogenic processes, they leave healthy microbiota largely intact [18]. While numerous antivirulence drug candidates have been documented [23], only three antivirulence therapeutics have gained US Food and Drug Administration (FDA) approval, all of which are antibody-based [9]. This suggests an emerging opportunity for the development of small molecule antivirulence drugs. While high-throughput screening (HTS) has been a mainstay of the pharmaceutical industry, its success rate has been extremely low in the field of antimicrobial development [24]. As structural information for validated virulence targets becomes more available, structure-based drug design (SBDD) is poised to become the new frontier in the antivirulence field, offering new possibilities to push antivirulence therapeutics into the mainstream. Here, we examine the application of SBDD to the field of antivirulence drug development. 

## 2. Principles of Structure-Based Drug Design

### 2.1. Rationale and Target Selection

SBDD is a powerful tool that uses knowledge of the three-dimensional (3D) structure of a biological target to efficiently search chemical space for ligands with high binding affinity. Use of SBDD began in the mid-1980s, and publications describing its success in developing new therapeutics for HIV/AIDS began to emerge in the early 1990s [25,26]. Structural knowledge of the HIV protease allowed for the design of new protease inhibitors, five of which became clinical and commercial successes [26,27]. Many other widely-used drugs, including zanamivir (Relenza; GlaxoSmithKline) [28] for influenza, the cyclooxygenase inhibitor celecoxib (Celebrex; Pfizer) [29], and the antileukemic Bcr-Abl tyrosine kinase inhibitor imatinib (Gleevec; Novartis) [30], owe their origins to SBDD. 

SBDD begins with the choice of a suitable target. An antivirulence target should be present only in the pathogen, and be essential for bacterial pathogenesis but not directly involved in bacterial survival. The vast majority of antivirulence targets are proteins, although RNA molecules may also be targeted, as is the case for the well-known antibiotics tetracycline and streptomycin [31]. The target should ideally be validated in vivo by testing a knockout strain of the bacteria for virulence in an animal model of infection. Accurate structural information about the target must then be obtained. Three main sources of structural information have been used for SBDD: X-ray crystallography, nuclear magnetic resonance (NMR), and homology modelling. X-ray crystal structures are the most common source of drug design data, owing to the typically high resolution available and the ability to work with proteins that range in size from small peptides to 998 kDa [32]. Indeed, 83.8% of the total protein structures in the Protein Data Bank (PDB) as of December 2018 were determined via X-ray crystallography. Ordered water molecules are also visible in crystal structures, the organization of which can often provide a starting point for drug lead design. NMR structures are another valuable source for drug design, provided that the target is smaller than 35 kDa [33]. The low resolution of structures gained by cryo-electron microscopy (EM) has precluded their use in SBDD in the past, however, best recent cryo-EM structures have surpassed the ~2.5 Å atomic resolution threshold and may thus be useful for drug design in the future [34]. Experimentally determined structures are curated in the PDB, which currently contains over 83,000 bacterial protein entries out of more than 145,000 total entries. For cases in which no experimentally determined structure is available, a homology model can be used for drug design provided there is substantial sequence similarity between the proteins [35,36]. Advanced homology modeling software uses experimentally determined structures as a template to predict the 3D-folds of another protein that has similar amino acid sequence [37]. Several free programs, including SWISS-MODEL [38] and Phyre2 [39] provide fully automated homology modeling. Commercial programs such as Modeller [40] or the modelling tools built into software suites such as Discovery Studio (BIOVIA) [41] offer additional control over the modelling process. Although homology models are routinely used in the absence of experimentally determined structures, it is noted that they are not ideal for SBDD since the accuracy of the binding pocket can be less reliable, particularly when sequence identity is below 40% [37].

The next step in SBDD involves using the available structure or model to select a specific ligand binding site. The target usually has a well-defined binding pocket, such as a receptor ligand binding site or enzyme active site. Algorithms are now available to predict the suitability of a binding pocket based on criteria such as its rigidity or hydrophobic character as calculated from high-resolution protein structures [42,43]. Although much less common, drugs targeting protein-protein interactions (PPIs) are increasingly being pursued by drug discovery groups [44]. An important, but often overlooked, aspect of target choice is the conformational flexibility of a protein during ligand binding. While the structure of the protein in the crystal represents a snapshot ‘frozen’ in one position, the biologically active form of the protein may undergo dramatic conformational changes upon ligand binding. This highlights the potential importance of being able to model protein and ligand flexibility in SBDD. Several programs such as GOLD [45], SLIDE [46] and FlexE [47] can be used to this end, however, the increased computing time required can be prohibitive [48]. Additionally, these programs only account for side chain flexibility, which can be insufficient when modelling more complex protein backbone motions as in the case of receptors [49].

### 2.2. Methods of SBDD

**Figure 2:**
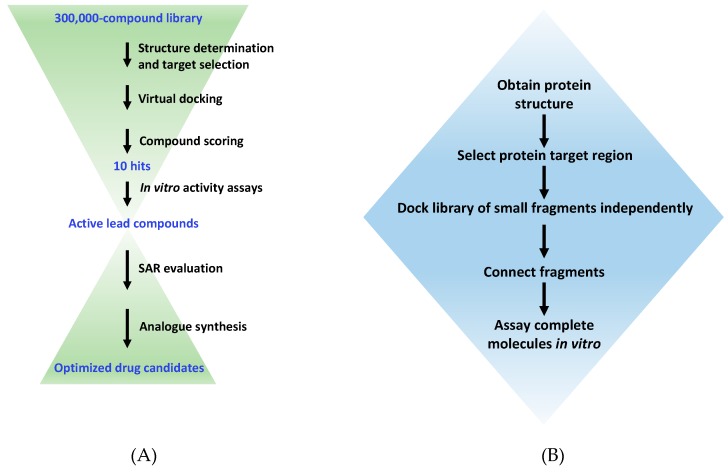
A typical workflow for virtual high-throughput screening and de novo drug design. (**A**) The virtual screening procedure used to select active compounds from a large library of existing molecules. SAR, structure-activity relationship. (**B**) The procedure for de novo design of ligands via the fragment-based approach using programs such as LUDI or SPROUT.

Once a target has been selected, there are three main methods that are used to identify or design new ligands based on its structural information: Inspection of substrate and known inhibitors, virtual screening, and de novo design. In the first method, inspection of substrates, cofactors, or known inhibitors of the protein is used to inform the modification of these compounds to become inhibitors [25,50,51]. In virtual screening, libraries of available small molecules are docked into the region of interest in silico and scored based on their predicted interaction with the site. The third approach involves de novo design of small molecule fragments that are positioned in the target site, scored, and then linked in silico to give one complete molecule (Figure 2). The final linked compounds are then chemically synthesized and tested for biological activity. Recent examples of these three approaches in the context of antivirulence drug discovery are discussed below. 

## 3. Substrate- and Known Inhibitor-Based Design

**Figure 3:**
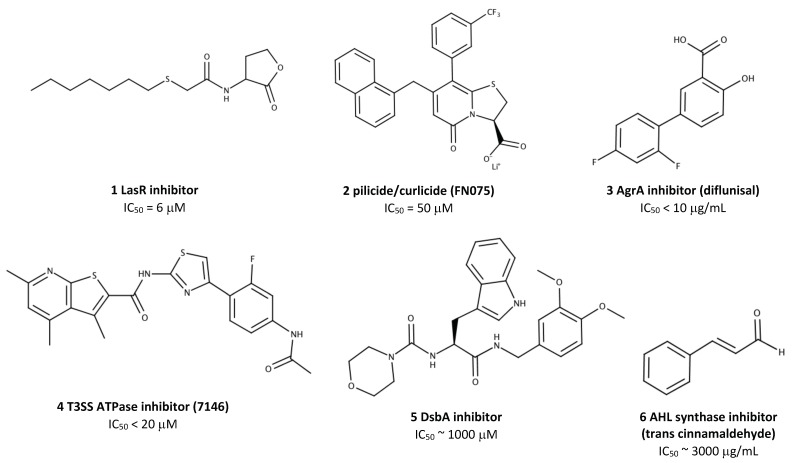
The structures of antivirulence compounds that have been discovered using SBDD techniques. IC_50_, half-maximal inhibitory concentration. 1, LasR quorum sensing inhibitor N-(heptylsulfanylacetyl)-L-homoserine lactone [54]; 2, bifunctional pilicide/curlicide FN075 [61]; 3, AgrA transcription factor inhibitor diflunisal [81]; 4, Type 3 secretion system (T3SS) inhibitor 7146 [74]; 5, DsbA thiol disulfide oxidoreductase inhibitor [85]; 6, acetyl homoserine lactone (AHL) synthase quorum sensing inhibitor trans cinnamaldehyde [89].

It is frequently useful to take the first cue in drug design from Nature. When a substrate or cofactor of a target protein is known, it can be structurally modified to create natural-product based inhibitors [52]. Indeed, the HIV protease inhibitors that began the SBDD revolution were themselves based on the unique Phe-Pro and Tyr-Pro cleavage site motifs present in the HIV polyprotein substrates [26]. Typically, the process begins with a co-crystal structure of the target protein in complex with the substrate, cofactor, binding partner, or drug lead. Modifications are then made based on the interactions with the target site to increase inhibitor potency or improve pharmacokinetic properties. Substrate modification has been applied to quench quorum sensing, which is a population density-dependent mode of communication used by pathogenic bacteria to coordinate virulence via the production and detection of acyl homoserine lactone (AHL) signal molecules (Figure 1). A potent quorum sensing inhibitor was derived from an endogenous *Pseudomonas aeruginosa* AHL by exchanging the hexanone ring for a phenolic ring, resulting in downstream inhibition of elastase virulence factor production [53]. Later, the complex structure of AHL with its cognate response regulator TraR from *Agrobacterium tumefaciens* guided the design of the potent quorum sensing inhibitor N-(heptylsulfanylacetyl)-L-homoserine lactone [54] (molecule 1, Figure 3). 

Peptidomimetics also fall under the umbrella of substrate-inspired drug design. These small (<2 kDa) peptide-like fragments mimic the interaction interface between larger proteins, thereby outcompeting the endogenous binding interaction. Unlike small molecules, peptidomimetics are particularly effective for disrupting protein-protein interaction as they can occupy the large surface area of a binding cleft [55]. Peptidomimetics have been successfully demonstrated to inhibit bacterial secretion systems, which are used by Gram-negative species to transport virulence factors through the normally-impermeable outer membrane (Figure 1). Synthetic peptides mimicking the coiled-coil regions of the translocator protein EspA and the needle protein EscF of the *Escherichia coli* type 3 secretion system (T3SS) effectively inhibited the T3SS dependent hemolysis of red blood cells [56]. Our group recently demonstrated the first structure-guided inhibition of the type 2 secretion system (T2SS) in *P. aeruginosa* using peptidomimetics. The synthetic peptides targeted the interface between XcpV-XcpW, which form a core binary complex in the secretion pseudopilus as determined via protein complex structures. The peptides inhibited toxin secretion in vitro and significantly attenuated the virulence of *P. aeruginosa* in a *Caenorhabditis elegans* model of infection [57], further validating the feasibility of targeting virulence pathways to treat bacterial infections. 

While peptidomimetics can provide an important proof-of-principle in the laboratory, their practical use in the clinic is severely limited by poor bioavailability, low stability, rapid degradation and clearance from the blood [58]. Small molecules that incorporate only the key structural elements of larger peptidomimetics are thus an attractive alternative, as demonstrated in the case of chaperone-usher pathway inhibitors, termed pilicides. These molecules inhibit the assembly of adhesive pili, which are used by Gram-negative pathogens for binding, invasion, and biofilm formation on epithelial cells (Figure 1). Pilicides were designed based on structural studies of the chaperone PapD and its cognate pilus subunits, which revealed a critical Arg8 and Lys112 cleft in the chaperone with which pilus subunits normally interact [59]. Small molecule mimetics of the C-terminus of the pilus subunits effectively disrupted pilus-dependent virulence phenomena, including bacterial attachment and biofilm formation [59,60]. This original pilicide has since been modified to inhibit curli biofilm biogenesis via a similar mechanism, resulting in attenuated virulence of *E. coli* in murine bladder infection [61] (molecule 2, Figure 3).

## 4. Virtual Screening of Chemical Libraries

### 4.1. Background

In silico docking enables rapid screening of very large collections of molecules—up to 100,000 per day when using a cluster of parallel computers [62]. The docked molecules typically originate from a large public database such as the National Institutes of Health Clinical Collection, from which hit molecules can readily be obtained for testing in biochemical assays. Many compounds are also available from commercial sources. At the core of each docking program is an algorithm that scores the validity of docking poses. Shape, van der Waals forces, electrostatic interactions, solvent accessible surface area and the formation of hydrogen bonds are all approximated by a docking score. A detailed evaluation of the computational methods used in virtual screening will not be provided here, as this has been extensively reviewed elsewhere [49]. Different algorithms place different weightings on each binding metric, thus a consensus scoring approach is recommended. If the same compound is predicted to bind tightly by multiple algorithms, this molecule can be more confidently selected as a lead. 

Docking in its simplest form began with rigid-body systems, where both protein and ligand assume fixed conformations and ligand binding poses are searched in six-dimensional space via rotation or translation [63]. Many reports have emphasized the importance of accounting for protein and ligand flexibility in scoring, thus, most current software offers these options (Table 1). There are now more than 60 different docking programs available for academic and commercial use, reviewed in depth elsewhere [49,64,65,66]. AutoDock, GLIDE (Schrödinger) and GOLD (The Cambridge Crystallographic Data Centre, Cambridge, UK) are three predominant programs whose application in antivirulence drug discovery will be discussed below. 

### 4.2. AutoDock

AutoDock is a free suite of docking programs that is used to predict how a small, flexible ligand will bind to a target of known 3D structure. The AutoDockTools front-end graphical interface is used to set up and analyze a docking experiment. Then ‘autodock’ performs the docking calculations of the ligand to the target protein, which is represented by a set of grids pre-calculated by ‘autogrid’. Older versions of the software (versions 2.4-4.0) use Monte Carlo simulated annealing to search ligand conformations, and a grid-based approach for energy evaluation [67]. A genetic algorithm is used to generate a series of docking poses and cluster them based on energy similarity. Interestingly, studies have shown that it is the most populated cluster, and not necessarily the lowest-energy cluster that best-predicts the native state of the docked ligand [68,69,70]. AutoDock versions 4.0 and up incorporate the Assisted Model Building with Energy Refinement (AMBER) force field [71], along with free energy scoring functions based on known inhibition constants for a large sample of protein-ligand complexes. 

An offshoot of the original program, called AutoDock Vina, eliminated the empirical scoring function and the genetic algorithm energy clustering of former versions in favour of knowledge-based scoring. This is accomplished via Monte Carlo sampling and the Broyden-Fletcher-Goldfarb-Shanno method for local optimization. AutoDock Vina yielded significant improvement in both prediction accuracy and docking time [72]. PSOVina is a particle swarm optimization derivative of the AutoDock Vina framework that dramatically reduces execution time without compromising docking accuracy [73], highlighting the potential for swarm intelligence in screening. 

An example of the application of AutoDock to antivirulence drug design is the structure-based screening of the ZINC chemical database using a Phyre homology model of the YscN ATPase, a key enzyme in the *Yersinia pestis* T3SS [74]. Docking results were obtained via the DOVIS large-scale virtual screening pipeline, using AutoDock 4.0 as the engine. YscN was modeled as a rigid-body molecule, and the ligands were allowed to be flexible. The average free energy of ligand binding was calculated for each cluster, in order to down-select the top 20,000 compounds from the sampled 5 million. Further minimization using CHARMm and re-scoring with LigScore2 narrowed the lists to 50 compounds, which were tested in vitro. Several best-performing compounds inhibited YscN ATPase activity at concentrations below 50 μM. These compounds were also effective at blocking T3SS-mediated YopE secretion by *Y. pestis*, and one compound was able to fully inhibit *Y. pestis* cytotoxicity towards cultured mammalian cells at concentrations below 50 μM (molecule 4, Figure 3). The identified hit molecules also inhibited the homologous BsaS protein from *Burkholderia mallei* [74].

### 4.3. Glide

Grid-based LIgand Docking with Energetics (Glide) is another popular docking program offered by Schrödinger [76]. Glide performs a near-exhaustive search of the positional, orientational, and conformational space available to the ligand by using a series of hierarchical filters. As with AutoDock, target proteins are represented by a pre-computed grid. Next, a set of initial ligand poses is selected from an exhaustive search of the minima in the ligand torsion-angle space. The poses selected from this initial screen are then minimized according to molecular mechanics and a distance-dependent dielectric model. A Monte Carlo procedure uses nearby torsional minima to properly orient peripheral groups, then, an empirical GlideScore is computed [76]. The GlideScore approximates ligand binding free energy by rewarding and penalizing a combination of many terms known to influence ligand binding, including electrostatic and van der Waals forces. Glide has been used to discover several effective antivirulence molecules, including inhibitors of *Staphylococcus aureus* response regulator AgrA [81] and the *P. aeruginosa* quorum sensing receptors LasR and RhlR [82]. 

*S. aureus* AgrA is a transcription factor for the expression of several predominant toxins and virulence factors that mediate the pathogenesis of this bacterium. Specifically, the toxins alpha-hemolysin (Hla) and phenol-soluble modulin α damage host cell membranes to facilitate bacterial evasion of host defences [83]. Expression of these toxins involves a complex quorum sensing cascade, a key step of which is the phosphorylation and subsequent dimerization of the N-terminal regulatory domain of AgrA. As no crystal structure of the N-terminal regulatory domain of *S. aureus* AgrA was available, a homology model was constructed via the Swiss-Model server based on the regulatory domain of the sigma 54 transcriptional activator NtrC1 from *Aquifex aeolicus*. Glide was used to dock compounds from the National Cancer Institute library of 90,000 small molecules. Compounds were docked to a grid covering a 10 Å cube centered on the phosphoryl acceptor residue Asp 59. The 107 top-scoring compounds were tested in vitro. At 10 μg/mL, four compounds inhibited Hla secretion by more than 70%, in addition to markedly decreasing hemolysis of rabbit erythrocytes. Interestingly, one of the top four compounds was the FDA-approved nonsteroidal anti-inflammatory drug diflunisal [81] (molecule 3, Figure 3), presenting an opportunity for off-label testing as an antivirulence prophylactic or adjuvant in humans. 

In another study, Glide was used to dock 1,920 natural compounds against the *P. aeruginosa* LasR and RhlR quorum sensing receptors [82]. These receptors bind AHL signalling molecules (as discussed in Section 3), causing conformational changes that allow DNA binding and transcriptional activation of downstream virulence genes (Figure 1). Quorum sensing controls approximately 350 genes in *P. aeruginosa*, of which ~30% encode virulence factors [84]. The crystal structure of LasR (PDB ID: 2UV0) was used for docking, while a homology model was used for RhlR. Grid generation was performed around the ligand binding site and ligands were docked flexibly via the standard Glide protocol. Four top-scoring compounds, namely rosmarinic acid, naringin, chlorogenic acid, and morin significantly inhibited the production of protease, elastase and hemolysin at concentrations between 750 to 1000 μg/mL. Other virulence determinants, such as biofilm formation and swarming motility, were also attenuated by the compounds [82]. 

### 4.4. GOLD

GOLD stands for Genetic Optimization for Ligand Docking. Unlike Glide, which treats the target protein as rigid, GOLD treats the target protein side-chains as flexible. This ability affords GOLD a more realistic and customizable docking methodology, with a wide range of constraints available to tailor docking towards a known motif or critical H-bond interaction. Like AutoDock, GOLD uses a genetic algorithm to probe ligand flexibility. This involves an evolutionary process whereby ligand geometry parameters are mapped onto ′chromosomes′, which are then subjected to iterative rounds of mutation, crossover, scoring and selection to optimize binding orientation. GOLD also scores the displacement of loosely bound water molecules upon ligand binding, which is a fundamental requirement prior to forming a new H-bond with a drug [45]. In general, GOLD excels with hydrophilic targets with some lipophilic character in the active site, but it performs significantly worse than Glide when binding is mainly mediated by hydrophobic contacts [49]. 

In one study using GOLD, a peptidomimetic fragment library was screened for inhibitors of DsbA [85]. This dithiol oxidoreductase protein is a key component of the oxidative folding system in Gram-negative bacteria, and plays a particularly important role in virulence factor assembly in the periplasm prior to secretion (Figure 1). Bacterial strains lacking Dsb enzymes are viable, but avirulent [86]. The 1.6-Å resolution crystal structure of *Proteus mirabilis* DsbA in complex with a DsbB-based hexapeptide inhibitor (PDB ID: 4OD7) [87] was used as a starting point to screen for more ‘drug-like’ compounds in silico. One initial hit molecule scored well using three different scoring methods available in GOLD (GoldScore, ChemScore, and ChemPLP), and was thus selected for further modification. The derivative with two additional methoxy groups on the phenyl ring of the lead yielded the best activity (molecule 5, Figure 3), with an IC_50_ of ~1 mM as measured by synthetic substrate fluorescence [85]. This activity is ~200 fold lower than that observed for larger DsbB peptidomimetics [88], indicating that DsbA may not be an ideal candidate for small molecule inhibition. 

GOLD has also been applied to elucidate the mechanism of binding of known quorum quenching molecules [89]. Using a reporter strain of *E. coli* that expressed the functional AHL synthases LasI and RhlI of *P. aeruginosa*, pure compounds of plant origin were screened for AHL synthase inhibition. The most potent inhibitor, trans-cinnamaldehyde (molecule 6, Figure 3), was then docked to a LasI crystal structure. Based on the docking pose, the authors postulated that trans-cinnamaldehyde forms hydrophobic and Pi-Pi interactions with Phe27, Trp33 and Phe105, and one H-bond with Arg30 of LasI. These residues are completely conserved, and form the putative binding pocket for *S*-adenosylmethionine—the source of the homoserine lactone ring required for AHL synthesis [89]. The orientation of the bound inhibitor will help inform its chemical modification to improve affinity, perhaps exploiting the well-conserved acyl-chain binding tunnel [90]. Similarly, another study used Gold scores to identify *N*-acetyl glucosamine as an inhibitor of LasR, TraR, and CviR quorum sensing receptors [91].

## 5. De Novo Ligand Design Based on Protein Structure

### 5.1. Background

De novo ligand design is the most recent addition to the SBDD toolkit. Since its inception in the early 1990s, de novo design has seen significant improvement owing to increasing computational power and improved algorithms. Most of the de novo design programs follow a similar operation pipeline: small functional groups are docked into the target site, scored, and then linked to form a whole molecule (Table 1). Since de novo SBDD involves docking fragments of molecules, this technique is synonymously referred to as fragment-based design. Common programs include LUDI (Discovery Studio, BIOVIA, San Diego, CA, USA; discussed below), SPROUT (Keymodule Ltd., Leeds, UK), and MCSS (Schrödinger LLC, New York, NY, USA). While de novo design can lead to the creation of novel compounds, this also means that costly custom synthesis of compounds is required for in vitro validation. Current versions of LUDI and other programs now incorporate algorithms to predict the synthetic feasibility of new compounds, thus ensuring only practical molecules are pursued. De novo methods have been used to design several antivirulence drugs.

### 5.2. LUDI

LUDI builds new ligands for the cleft of a given protein of known 3D structure. This program uses rules about energetically favourable nonbonded contacts to score interactions between specific moieties of the protein and ligand. These rules are statistically derived from analysis of the crystal packing observed in small organic structures from the Cambridge Structural Database (CSD). The program works in three steps to score and connect small fragments. First, interaction sites that are suitable to form hydrogen bonds or fill hydrophobic pockets are identified using the empirical data from the CSD. Small fragments from a library are then docked into the protein target site in such a way that H-bonds and ionic interactions with the protein are maximized, and lipophilic groups on the ligands fill hydrophobic pockets in the protein. The final step involves appending further fragments, some or all of which are connected to form a complete molecule [78]. The newly-connected putative ligands then receive an overall binding score using a simple algorithm based on empirical binding constants of protein-ligand complexes [92]. 

**Figure 4:**
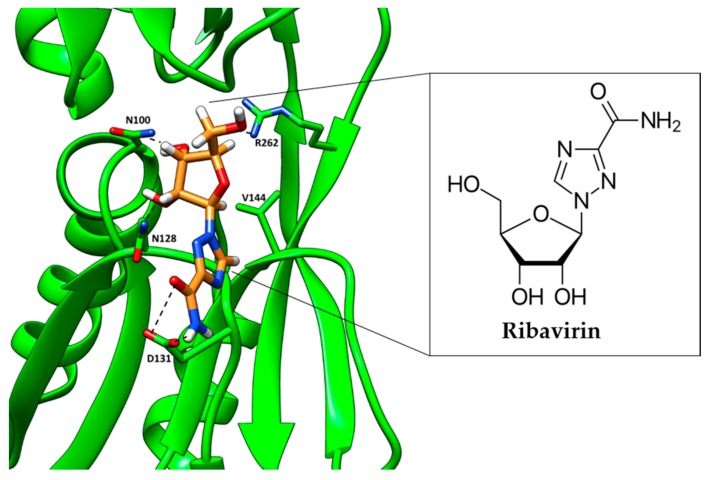
An inhibitor of AphB (ribavirin), discovered using LUDI, docked in the co-inducer-binding site of *V. cholerae* AphB (PDB ID 3SZP) [93]. The inhibitor interacts with key labeled residues; H-bonding is shown by dashed lines. Ribavirin is shown in orange, and the AphB backbone is shown as green ribbons.

Recently, LUDI was used to target AphB, a *Vibrio cholerae* LysR-type transcriptional regulator that regulates the expression of genes encoding cholera toxin and the toxin-co-regulated pilus [93]. The crystal structure of AphB from *V. cholerae* (PDB ID: 3SZP) was used for inhibitor design. 1491 commercially-available fragments were extracted from a database of FDA approved drugs and screened in LUDI. Fragments were selected based on critical interactions with residues in the active site of AphB and the Gibbs free energy of binding. Three top-scoring fragments were used as the basis for multiple linked-fragment molecules. The resulting novel scaffolds were then subjected to a substructure search in the PubChem database, which identified 1087 drug-like small molecules. These molecules were docked using Glide XP. Among the top seven hits was ribavirin, an FDA-approved antiviral drug (Figure 4). In vitro testing via NMR and isothermal calorimetry confirmed ribavirin binding to AphB, with a dissociation constant ~300 μM. Ribavirin at 100 μg/mL effectively inhibited cholera toxin production by cultured *V. cholerae*. Most impressively, ribavirin inhibited the intestinal colonization of *V. cholera*, significantly improving survival in a mouse model of infection [93]. 

Another virulence protein that has been targeted with LUDI is Staphylococcal accessory regulator A (SarA) [94]. This protein is a transcriptional activator of *S. aureus* quorum sensing, and it has been implicated in downstream biofilm formation and virulence protein secretion in this pathogen. The crystal structure of SarA (PDB ID: 2FRH) was processed, and the known active site comprised of three key residues (D88, E89 and R90) within a 7.7 Å radius sphere was screened for fragment binding. Fragments were then linked in LUDI and the resulting compounds were subject to CHARMm energy minimization, followed by docking with two separate programs to rank binding affinity [95]. The optimized fragment-based inhibitor 4-[(2,4-difluorobenzyl)amino] cyclohexanol was later synthesized and tested in vitro. At 200 μg/mL, this compound significantly inhibited *S. aureus* biofilm formation and adherence to HEp-2 cells, in addition to attenuating virulence in a rat model of medical implant infection [94].

### 5.3. De Novo Binding Protein Design

In addition to the de novo design of small molecule as outlined above, similar principles have been successfully applied to create small (4-12 kDa) binding proteins that bridge the gap between monoclonal antibodies and small molecule drugs. A recent study created a massively parallel de novo protein design pipeline that was successfully used to generate antivirulence proteins against *Clostridium botulinum* toxin (BoNT) [96]. First, a virtual library of over 4000 protein backbone geometries, with varying topologies and disulfide connectivities was generated in the Rosetta software suite. The helical segments of each backbone were then superimposed on the interface helices in the previously solved BoNT- B synaptotagmin-II co-crystal structure (B synaptotagmin-II is the natural target of BoNT). Hotspot residues from the endogenous complex were added to seed the helix of the binding protein. Rosetta combinatorial sequence optimization was then used to design the remaining residues to maximize binding affinity and stability. Yeast display and deep sequencing were used to test and identify successful binding proteins. Several proteins bound to botulinum toxin with 1–20 nM affinity and protected rat cortical neurons from entry and damage by the toxin [96].

## 6. Conclusions and Outlook

As the global burden of bacterial resistance to traditional antibacterial drugs continues to rise, the need for new alternatives is becoming increasingly dire. In some cases, clinical isolates have been described that are resistant to all previously effective antimicrobial agents [97]. The high throughput screening programs that have dominated the search for new antibacterial drugs have failed to deliver, and a consensus has emerged that new approaches are needed [98]. The development of new drugs targeting virulence pathways is one such approach, which has seen a steady increase in research activity over the past decade [18]. Likewise, the use of SBDD in all fields of pharmaceutical development experienced a similar burgeoning in recent years. The increasing availability of high-resolution structures of virulence proteins, along with faster, more accurate computer docking and design programs, have coalesced to facilitate the rational design of antivirulence compounds as described in this review. While the plethora of druggable virulence targets offers exciting prospects for new pharmaceuticals, more basic research into virulence pathways, models of virulence, and how to minimize the development of antivirulence drug resistance is required. Evaluating the effectiveness of antivirulence drugs requires conditions that more closely resemble the pathogen’s physiological niche, in contrast to the straightforward growth inhibition assays used for traditional antibiotics. This will require greater up-front investment from pharmaceutical companies, which may be a difficult proposition in an industry already weary of antimicrobial drug profitability. However, the field of antivirulence research is still in its infancy, and will undoubtedly continue to grow in future years. In the post-antibiotic era, rationally-designed antivirulence drugs have the potential to become an indispensable weapon against bacterial infection.

## Figures and Tables

**Table 1:** A comparison of widely used SBDD computer programs.

	Program	Search strategy	Flexible ligand?	Flexible protein side chains?	Description	Free for academia?
**Virtual screening**	AutoDock [67,74]	GA/MC	yes	yes	Exhaustive search of interaction energy grid followed by simulated annealing energy scoring	yes
	GOLD [45]	GA	yes	yes	Positions ligand and minimizes energy via an evolutionary algorithm	no
	eHITS [75]	IC	yes	no	Ligands are divided into rigid fragments, which are docked individually, then reconstructed	no
	GLIDE [76]	Hybrid	yes	no	Protein-ligand coulomb-vdW energy minimization and an empirical GlideScore	no
	FlexE [47]	IC	yes	yes	Incremental construction by sampling ligand conformations and target ensembles	no
	ICM [77]	MC	yes	yes	Monte-Carlo energy minimization of protein and ligand	no
**De novo ligand design**	LUDI [78]	EG	yes	no	Empirically-derived energy scoring of fragments	no
	SPROUT [79]	AI	yes	no	Template atoms or fragments are linked to make skeletons	no
	MCSS [80]	Hybrid	yes	no	CHARMm-based exhaustive search for target site functional group minima	no

GA, genetic algorithm; MC, Monte-Carlo, IC, incremental construction; EG, empirical geometry; AI, artificial intelligence; FF, force field.

## References

[1] Chopra I., Hesse L., O’Neill A.J. (2002). Exploiting current understanding of antibiotic action for discovery of new drugs. J. Appl. Microbiol..

[2] Knowles D.J.C. (1997). New strategies for antibacterial drug design. Trends Microbiol..

[3] White A.R. (2011). BSAC Working Party on The Urgent Need: Regenerating Antibacterial Drug Discovery and Development Effective antibacterials: At what cost? The economics of antibacterial resistance and its control. J. Antimicrob. Chemother..

[4] Kmietowicz Z. (2017). Few novel antibiotics in the pipeline, WHO warns. BMJ.

[5] Spellberg B., Guidos R., Gilbert D., Bradley J., Boucher H.W., Scheld W.M., Bartlett J.G., Edwards J., Infectious Diseases Society of America (2008). The epidemic of antibiotic-resistant infections: A call to action for the medical community from the Infectious Diseases Society of America. Clin. Infect. Dis..

[6] Talbot G.H., Bradley J., Edwards J.E., Gilbert D., Scheld M., Bartlett J.G. (2006). Bad bugs need drugs: An update on the development pipeline from the Antimicrobial Availability Task Force of the Infectious Diseases Society of America. Clin. Infect. Dis..

[7] Chopra I., Schofield C., Everett M., O’Neill A., Miller K., Wilcox M., Frère J.-M., Dawson M., Czaplewski L., Urleb U. (2008). Treatment of health-care-associated infections caused by Gram-negative bacteria: A consensus statement. Lancet Infect Dis.

[8] Ventola C.L. (2015). The Antibiotic Resistance Crisis. P T.

[9] Dickey S.W., Cheung G.Y.C., Otto M. (2017). Different drugs for bad bugs: Antivirulence strategies in the age of antibiotic resistance. Nat. Rev. Drug Discov..

[10] Spees A.M., Wangdi T., Lopez C.A., Kingsbury D.D., Xavier M.N., Winter S.E., Tsolis R.M., Bäumler A.J. (2013). Streptomycin-Induced Inflammation Enhances Escherichia coli Gut Colonization Through Nitrate Respiration. mBio.

[11] Maura D., Ballok A.E., Rahme L.G. (2016). Considerations and caveats in anti-virulence drug development. Curr. Opin. Microbiol..

[12] Falkow S. (1991). What is a pathogen?. Am. Soc. Microbiol. News.

[13] Keller M.A., Stiehm E.R. (2000). Passive immunity in prevention and treatment of infectious diseases. Clin. Microbiol. Rev..

[14] Schmitt C.K., Meysick K.C., O’Brien A.D. (1999). Bacterial toxins: Friends or foes?. Emerg. Infect. Dis..

[15] Cegelski L., Marshall G.R., Eldridge G.R., Hultgren S.J. (2008). The biology and future prospects of antivirulence therapies. Nat. Rev. Microbiol..

[16] Rasko D.A., Sperandio V. (2010). Anti-virulence strategies to combat bacteria-mediated disease. Nat. Rev. Drug Discov..

[17] Escaich S. (2008). Antivirulence as a new antibacterial approach for chemotherapy. Curr. Opin. Chem. Biol..

[18] Totsika M. (2016). Benefits and Challenges of Antivirulence Antimicrobials at the Dawn of the Post-Antibiotic Era. Curr. Med. Chem..

[19] Kalia V.C. (2013). Quorum sensing inhibitors: An overview. Biotechnol. Adv..

[20] Baron C. (2010). Antivirulence drugs to target bacterial secretion systems. Curr. Opin. Microbiol..

[21] Cusumano C.K., Hultgren S.J. (2009). Bacterial adhesion—A source of alternate antibiotic targets. IDrugs.

[22] Totsika M. (2017). Disarming pathogens: Benefits and challenges of antimicrobials that target bacterial virulence instead of growth and viability. Future Med. Chem..

[23] Zambelloni R., Marquez R., Roe A.J. (2015). Development of Antivirulence Compounds: A Biochemical Review. Chem. Biol. Drug Des..

[24] Overbye K.M., Barrett J.F. (2005). Antibiotics: Where did we go wrong?. Drug Discov. Today.

[25] Erickson J., Neidhart D.J., VanDrie J., Kempf D.J., Wang X.C., Norbeck D.W., Plattner J.J., Rittenhouse J.W., Turon M., Wideburg N. (1990). Design, activity, and 2.8 A crystal structure of a C2 symmetric inhibitor complexed to HIV-1 protease. Science.

[26] Roberts N.A., Martin J.A., Kinchington D., Broadhurst A.V., Craig J.C., Duncan I.B., Galpin S.A., Handa B.K., Kay J., Kröhn A. (1990). Rational design of peptide-based HIV proteinase inhibitors. Science.

[27] Dorsey B.D., Levin R.B., McDaniel S.L., Vacca J.P., Guare J.P., Darke P.L., Zugay J.A., Emini E.A., Schleif W.A. (1994). L-735,524: The Design of a Potent and Orally Bioavailable HIV Protease Inhibitor. J. Med. Chem..

[28] McCauley J. (1999). Relenza. Curr. Biol..

[29] Stratton M.S., Alberts D.S. (2002). Current application of selective COX-2 inhibitors in cancer prevention and treatment. Oncology.

[30] Deininger M., Buchdunger E., Druker B.J. (2005). The development of imatinib as a therapeutic agent for chronic myeloid leukemia. Blood.

[31] Hong W., Zeng J., Xie J. (2014). Antibiotic drugs targeting bacterial RNAs. Acta Pharm. Sin. B.

[32] Nissen P., Hansen J., Ban N., Moore P.B., Steitz T.A. (2000). The Structural Basis of Ribosome Activity in Peptide Bond Synthesis. Science.

[33] Yu H. (1999). Extending the size limit of protein nuclear magnetic resonance. Proc. Natl. Acad. Sci. USA.

[34] Bartesaghi A., Merk A., Banerjee S., Matthies D., Wu X., Milne J.L.S., Subramaniam S. (2015). 2.2 Å resolution cryo-EM structure of β-galactosidase in complex with a cell-permeant inhibitor. Science.

[35] Vyas V.K., Ukawala R.D., Ghate M., Chintha C. (2012). Homology Modeling a Fast Tool for Drug Discovery: Current Perspectives. Indian J. Pharm. Sci..

[36] Enyedy I.J., Lee S.L., Kuo A.H., Dickson R.B., Lin C.Y., Wang S. (2001). Structure-based approach for the discovery of bis-benzamidines as novel inhibitors of matriptase. J. Med. Chem..

[37] Xiang Z. (2006). Advances in Homology Protein Structure Modeling. Curr. Protein Pept. Sci..

[38] Arnold K., Bordoli L., Kopp J., Schwede T. (2006). The SWISS-MODEL workspace: A web-based environment for protein structure homology modelling. Bioinformatics.

[39] Kelley L.A., Mezulis S., Yates C.M., Wass M.N., Sternberg M.J. (2015). The Phyre2 web portal for protein modelling, prediction and analysis. Nat. Protoc..

[40] Webb B., Sali A. (2014). Comparative Protein Structure Modeling Using MODELLER. Curr. Protoc. Bioinformatics.

[41] Kemmish H., Fasnacht M., Yan L. (2017). Fully automated antibody structure prediction using BIOVIA tools: Validation study. PLoS ONE.

[42] Halgren T.A. (2009). Identifying and Characterizing Binding Sites and Assessing Druggability. J. Chem. Inf. Model..

[43] Villoutreix B.O., Renault N., Lagorce D., Sperandio O., Montes M., Miteva M.A. (2007). Free resources to assist structure-based virtual ligand screening experiments. Curr. Protein Pept. Sci..

[44] Scott D.E., Bayly A.R., Abell C., Skidmore J. (2016). Small molecules, big targets: Drug discovery faces the protein–protein interaction challenge. Nat. Rev. Drug Discov..

[45] Jones G., Willett P., Glen R.C., Leach A.R., Taylor R. (1997). Development and validation of a genetic algorithm for flexible docking. J. Mol. Biol..

[46] Schnecke V., Swanson C.A., Getzoff E.D., Tainer J.A., Kuhn L.A. (1998). Screening a peptidyl database for potential ligands to proteins with side-chain flexibility. Proteins.

[47] Claussen H., Buning C., Rarey M., Lengauer T. (2001). FlexE: Efficient molecular docking considering protein structure variations. J. Mol. Biol..

[48] Simmons K.J., Chopra I., Fishwick C.W.G. (2010). Structure-based discovery of antibacterial drugs. Nat. Rev. Microbiol..

[49] Pagadala N.S., Syed K., Tuszynski J. (2017). Software for molecular docking: A review. Biophys. Rev..

[50] Chan D.C.M., Laughton C.A., Queener S.F., Stevens M.F.G. (2001). Structural Studies on Bioactive Compounds. 34. Design, Synthesis, and Biological Evaluation of Triazenyl-Substituted Pyrimethamine Inhibitors of Pneumocystis carinii Dihydrofolate Reductase. J. Med. Chem..

[51] Varney M.D., Marzoni G.P., Palmer C.L., Deal J.G., Webber S., Welsh K.M., Bacquet R.J., Bartlett C.A., Morse C.A. (1992). Crystal-structure-based design and synthesis of benz[cd]indole-containing inhibitors of thymidylate synthase. J. Med. Chem..

[52] Schmid M.B. (2004). Seeing is believing: The impact of structural genomics on antimicrobial drug discovery. Nat. Rev. Microbiol..

[53] Smith K.M., Bu Y., Suga H. (2003). Library Screening for Synthetic Agonists and Antagonists of a Pseudomonas aeruginosa Autoinducer. Chem. Biol..

[54] Persson T., Hansen T.H., Rasmussen T.B., Skindersø M.E., Givskov M., Nielsen J. (2005). Rational design and synthesis of new quorum-sensing inhibitors derived from acylated homoserine lactones and natural products from garlic. Org. Biomol. Chem..

[55] Akram O.N., DeGraff D.J., Sheehan J.H., Tilley W.D., Matusik R.J., Ahn J.-M., Raj G.V. (2014). Tailoring Peptidomimetics for Targeting Protein–Protein Interactions. Mol. Cancer Res..

[56] Larzábal M., Mercado E.C., Vilte D.A., Salazar-González H., Cataldi A., Navarro-Garcia F. (2010). Designed Coiled-Coil Peptides Inhibit the Type Three Secretion System of Enteropathogenic Escherichia coli. PLoS ONE.

[57] Zhang Y., Faucher F., Zhang W., Wang S., Neville N., Poole K., Zheng J., Jia Z. (2018). Structure-guided disruption of the pseudopilus tip complex inhibits the Type II secretion in Pseudomonas aeruginosa. PLoS Pathog..

[58] Qvit N., Rubin S.J.S., Urban T.J., Mochly-Rosen D., Gross E.R. (2017). Peptidomimetic therapeutics: Scientific approaches and opportunities. Drug Discov. Today.

[59] Pinkner J.S., Remaut H., Buelens F., Miller E., Aberg V., Pemberton N., Hedenström M., Larsson A., Seed P., Waksman G. (2006). Rationally designed small compounds inhibit pilus biogenesis in uropathogenic bacteria. Proc. Natl. Acad. Sci. USA.

[60] Lee Y.M., Almqvist F., Hultgren S.J. (2003). Targeting virulence for antimicrobial chemotherapy. Curr. Opin. Pharmacol..

[61] Cegelski L., Pinkner J.S., Hammer N.D., Cusumano C.K., Hung C.S., Chorell E., Åberg V., Walker J.N., Seed P.C., Almqvist F. (2009). Small-molecule inhibitors target Escherichia coli amyloid biogenesis and biofilm formation. Nat. Chem. Biol..

[62] Schnecke V., Kuhn L.A. (2000). Virtual screening with solvation and ligand-induced complementarity. Perspect. Drug Discov. Des..

[63] Alberg D.G., Schreiber S.L. (1993). Structure-based design of a cyclophilin-calcineurin bridging ligand. Science.

[64] Taylor R.D., Jewsbury P.J., Essex J.W. (2002). A review of protein-small molecule docking methods. J. Comput. Aided Mol Des.

[65] Shoichet B.K., McGovern S.L., Wei B., Irwin J.J. (2002). Lead discovery using molecular docking. Curr. Opin. Chem. Biol..

[66] Ramírez D. (2016). Computational Methods Applied to Rational Drug Design. Open Med. Chem. J..

[67] Goodsell D.S., Morris G.M., Olson A.J. (1996). Automated docking of flexible ligands: Applications of AutoDock. J. Mol. Recognit..

[68] Prasad J.C., Goldstone J.V., Camacho C.J., Vajda S., Stegeman J.J. (2007). Ensemble modeling of substrate binding to cytochromes P450: Analysis of catalytic differences between CYP1A orthologs. Biochemistry.

[69] Källblad P., Mancera R.L., Todorov N.P. (2004). Assessment of Multiple Binding Modes in Ligand−Protein Docking. J. Med. Chem..

[70] Limongelli V., Marinelli L., Cosconati S., Braun H.A., Schmidt B., Novellino E. (2007). Ensemble-docking approach on BACE-1: Pharmacophore perception and guidelines for drug design. ChemMedChem.

[71] Case D.A., Cheatham T.E., Darden T., Gohlke H., Luo R., Merz K.M., Onufriev A., Simmerling C., Wang B., Woods R.J. (2005). The Amber Biomolecular Simulation Programs. J. Comput. Chem..

[72] Trott O., Olson A.J. (2010). AutoDock Vina: Improving the speed and accuracy of docking with a new scoring function, efficient optimization and multithreading. J. Comput. Chem..

[73] Ng M.C.K., Fong S., Siu S.W.I. (2015). PSOVina: The hybrid particle swarm optimization algorithm for protein-ligand docking. J. Bioinform. Comput. Biol..

[74] Swietnicki W., Carmany D., Retford M., Guelta M., Dorsey R., Bozue J., Lee M.S., Olson M.A. (2011). Identification of Small-Molecule Inhibitors of Yersinia pestis Type III Secretion System YscN ATPase. PLoS ONE.

[75] Zsoldos Z., Reid D., Simon A., Sadjad S.B., Johnson A.P. (2007). eHiTS: A new fast, exhaustive flexible ligand docking system. J. Mol. Graph. Model..

[76] Friesner R.A., Banks J.L., Murphy R.B., Halgren T.A., Klicic J.J., Mainz D.T., Repasky M.P., Knoll E.H., Shelley M., Perry J.K. (2004). Glide:  A New Approach for Rapid, Accurate Docking and Scoring. 1. Method and Assessment of Docking Accuracy. J. Med. Chem..

[77] Abagyan R., Totrov M., Kuznetsov D. (1994). ICM—A New Method for Protein Modeling and Design: Applications to Docking and Structure Prediction from the Distorted Native Conformation. J. Comput. Chem..

[78] Böhm H.J. (1992). The computer program LUDI: A new method for the de novo design of enzyme inhibitors. J. Comput. Aided Mol. Des..

[79] Gillet V., Johnson A.P., Mata P., Sike S., Williams P. (1993). SPROUT: A program for structure generation. J. Comput. Aided Mol. Des..

[80] Caflisch A., Miranker A., Karplus M. (1993). Multiple copy simultaneous search and construction of ligands in binding sites: Application to inhibitors of HIV-1 aspartic proteinase. J. Med. Chem..

[81] Khodaverdian V., Pesho M., Truitt B., Bollinger L., Patel P., Nithianantham S., Yu G., Delaney E., Jankowsky E., Shoham M. (2013). Discovery of Antivirulence Agents against Methicillin-Resistant Staphylococcus aureus. Antimicrob. Agents Chemother..

[82] Annapoorani A., Umamageswaran V., Parameswari R., Pandian S.K., Ravi A.V. (2012). Computational discovery of putative quorum sensing inhibitors against LasR and RhlR receptor proteins of Pseudomonas aeruginosa. J. Comput. Aided Mol. Des..

[83] Otto M. (2014). Staphylococcus aureus toxins. Curr. Opin. Microbiol..

[84] Rasmussen T.B., Givskov M. (2006). Quorum-sensing inhibitors as anti-pathogenic drugs. Int. J. Med. Microbiol..

[85] Duprez W., Bachu P., Stoermer M.J., Tay S., McMahon R.M., Fairlie D.P., Martin J.L. (2015). Virtual Screening of Peptide and Peptidomimetic Fragments Targeted to Inhibit Bacterial Dithiol Oxidase DsbA. PLoS ONE.

[86] Totsika M., Heras B., Wurpel D.J., Schembri M.A. (2009). Characterization of Two Homologous Disulfide Bond Systems Involved in Virulence Factor Biogenesis in Uropathogenic Escherichia coli CFT073. J. Bacteriol..

[87] Kurth F., Duprez W., Premkumar L., Schembri M.A., Fairlie D.P., Martin J.L. (2014). Crystal structure of the dithiol oxidase DsbA enzyme from proteus mirabilis bound non-covalently to an active site peptide ligand. J. Biol. Chem..

[88] Duprez W., Premkumar L., Halili M.A., Lindahl F., Reid R.C., Fairlie D.P., Martin J.L. (2015). Peptide inhibitors of the Escherichia coli DsbA oxidative machinery essential for bacterial virulence. J. Med. Chem..

[89] Chang C.-Y., Krishnan T., Wang H., Chen Y., Yin W.-F., Chong Y.-M., Tan L.Y., Chong T.M., Chan K.-G. (2014). Non-antibiotic quorum sensing inhibitors acting against N-acyl homoserine lactone synthase as druggable target. Sci. Rep..

[90] Gould T.A., Schweizer H.P., Churchill M.E.A. (2004). Structure of the Pseudomonas aeruginosa acyl-homoserinelactone synthase LasI. Mol. Microbiol..

[91] Kimyon Ö., Ulutürk Z.İ., Nizalapur S., Lee M., Kutty S.K., Beckmann S., Kumar N., Manefield M. (2016). N-Acetylglucosamine Inhibits LuxR, LasR and CviR Based Quorum Sensing Regulated Gene Expression Levels. Front. Microbiol..

[92] Boehm H.J., Boehringer M., Bur D., Gmuender H., Huber W., Klaus W., Kostrewa D., Kuehne H., Luebbers T., Meunier-Keller N. (2000). Novel inhibitors of DNA gyrase: 3D structure based biased needle screening, hit validation by biophysical methods, and 3D guided optimization. A promising alternative to random screening. J. Med. Chem..

[93] Mandal R.S., Ta A., Sinha R., Theeya N., Ghosh A., Tasneem M., Bhunia A., Koley H., Das S. (2016). Ribavirin suppresses bacterial virulence by targeting LysR-type transcriptional regulators. Sci. Rep..

[94] Arya R., Ravikumar R., Santhosh R.S., Princy S.A. (2015). SarA based novel therapeutic candidate against Staphylococcus aureus associated with vascular graft infections. Front. Microbiol..

[95] Arya R., Princy S.A. (2013). Computational approach to design small molecule inhibitors and identify SarA as a potential therapeutic candidate. Med. Chem. Res..

[96] Chevalier A., Silva D.-A., Rocklin G.J., Hicks D.R., Vergara R., Murapa P., Bernard S.M., Zhang L., Lam K.-H., Yao G. (2017). Massively parallel de novo protein design for targeted therapeutics. Nature.

[97] Rossi F., Girardello R., Cury A.P., Di Gioia T.S.R., de Almeida J.N., da Silva Duarte A.J. (2017). Emergence of colistin resistance in the largest university hospital complex of São Paulo, Brazil, over five years. Braz. J. Infect. Dis..

[98] Payne D.J., Gwynn M.N., Holmes D.J., Pompliano D.L. (2007). Drugs for bad bugs: Confronting the challenges of antibacterial discovery. Nat. Rev. Drug Discov..

